# Deficiency of SR‐B1 reduced the tumor load of colitis‐induced or APC^min^ ^/+^‐induced colorectal cancer

**DOI:** 10.1002/cam4.6534

**Published:** 2023-09-28

**Authors:** Qijun Chen, Lixue Wang, Hui Song, Wen Xing, Junfeng Shi, Yudi Li, Ziqian Wang, Jinlong Chen, Nan Xie, Wenhua Zhao

**Affiliations:** ^1^ School of Pharmaceutical Sciences Capital Medical University Beijing China; ^2^ School of Basic Medical Sciences Capital Medical University Beijing China

**Keywords:** colorectal cancer, intestinal immunity, intestinal microbiota, PD‐L1, scavenger receptor class B type 1

## Abstract

**Background:**

Colorectal cancer (CRC) is one of the most common tumors in the world. Cholesterol plays an important role in the pathogenesis of tumors. One of the cholesterol transporters, scavenger receptor class B type 1 (SR‐B1), a multi‐ligand membrane receptor protein, is expressed in the intestines which also highly expressed in various tumors. But the potential mechanism of SR‐B1 in CRC development has not been reported.

**Aims:**

This study aimed to clarify the importance of SR‐B1 in the development and prognosis of CRC as much as possible to provide a possible strategy in CRC treatment.

**Materials & Methods:**

In this study, we used SR‐B1 gene knockdown mice to study the effect of SR‐B1 on colitis‐induced or APC^min/+^‐induced CRC. The expression of related molecules were detected through the immunohistochemistry and hematoxylin–eosin staining, western blot analysis, and Flow cytometry. The gene expression and microbiota in microenvironment of CRC mice were analyzed through eukaryotic mRNA sequencing and 16S rRNA high‐throughput sequencing.

**Results:**

The results showed that SR‐B1 knockdown reduced the tumor load of colitis‐induced or APC^min/+^‐induced CRC. SR‐B1 knockdown improved the immune microenvironment by affecting the level of tumor‐associated macrophage (TAM), mononuclear myeloid‐derived suppressor cells (M‐MDSCs), granulocytic myeloid‐derived suppressor cells (G‐MDSCs), programmed cell death‐ligand 1 (PD‐L1), and human leukocyte antigen class I‐B (HLA‐B), and also reduced the level of low‐density lipoprotein receptor (LDL‐R), and increased the level of ATP binding cassette transporter A1 (ABCA1) to regulate the cholesterol metabolism, and regulated the expression of related genes and intestinal microbiota. SR‐B1 knockdown can also trigger the anti‐CRC effect of anti‐PD 1 in colitis‐induced CRC.

**Discussion:**

SR‐B1 deficiency significantly improved the immunity in tumor microenvironment of colitis‐induced or APC^min/+^‐induced CRC. In addition, the microbiota changes caused by SR‐B1 deficiency favor improving the immune response to chemotherapeutic drugs and anti‐PD1 therapy. The mechanism of action of SR‐B1 deficiency on the development of CRC still needs further in‐depth research.

**Conclusion:**

This study provides a new treatment strategy for treating CRC by affecting the expression of SR‐B1 in intestine.

## Graphical abstract

Scavenger receptor class B type 1 (SR‐B1) knockdown reduced the tumor load of colitis‐induced or APC^min/+^‐induced colorectal cancer (CRC). SR‐B1 knockdown improved the immune microenvironment and intestinal microbiota by controlling the tumor‐associated macrophage, mononuclear myeloid‐derived suppressor cells, granulocytic myeloid‐derived suppressor cells, and programmed cell death‐ligand 1 levels. SR‐B1 knockdown triggered the anti‐colon effect of anti‐PD 1 in CRC.

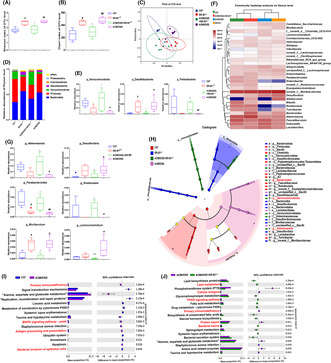

## 1 INTRODUCTION

Colorectal cancer (CRC) is one of the most common tumors in the world. The latest global statistics show that the incidence rate and mortality of CRC ranked third (10%) and second (9.4%) among all tumors.^1^ Colon and rectal cancer deaths are expected to increase by 60.0% and 71.5%, respectively, in all countries by 2035.^2^ Genetic and environmental risk factors play important roles in the development of CRC.^3^ In the process of disease progression, chronic stimuli lead to the gradual deterioration of the original normal colorectal mucosa, which forms CRC. Most CRC originated from precancerous lesions such as adenoma and then transformed into adenocarcinoma. At present, the best treatment for CRC is still radical surgery. However, due to the hiddenness in the early stage, most patients show symptoms in the late stage and cannot be treated by surgery. In addition, immune checkpoint inhibitors, including anti‐PD‐1 therapy, have limited efficacy in patients with CRC. The early molecular markers of CRC recurrence and metastasis tendency and intervention in clinical treatment can improve the prognosis. Therefore, it is urgent further to study the molecular biological mechanism of CRC progress, provide more effective clinical treatment and prevention methods for patients, and reduce the mortality of CRC. Recent studies have shown that cholesterol plays an important role in the pathogenesis of cancer. Cholesterol has been proven to regulate the basic signal pathways involved in cell proliferation, migration, and survival, thus promoting cancer progression.^4^ Tumor cells show a high affinity for cholesterol to support their inherent nature of division and proliferation. Scavenger receptor‐class B type 1 (SR‐B1), a high‐density lipoprotein (HDL) receptor,^5^ regulates the reverse transport of cholesterol.^6^, ^7^ In addition, that SR‐B1 is expressed in the intestine.^8^ SR‐B1 plays an important role in inducing endothelial cell migration,^9^ inhibiting macrophage apoptosis,^10^ and regulating lymphocyte autoimmunity and homeostasis.^11^ SR‐B1 deficiency can enhance lymphocyte proliferation, increase the production of inflammatory cytokines in macrophages, and reduce the inhibition of HDL on lymphocyte proliferation.^11^ SR‐B1 is highly expressed in breast cancer,^12^ prostate cancer, and nasopharyngeal carcinoma.^13^, ^14^ However, the potential role and molecular mechanism of SR‐B1 in CRC have not been reported. In this study, we used SR‐B1 gene knockdown mice to study the effect of SR‐B1 on colitis‐induced or APC^min/+^‐induced CRC.

## 2 MATERIALS AND METHODS

### 2.1 Animals

APC^min/+^ and SR‐B1^−/−^ mice were purchased from Shanghai Model Organisms Center, Inc. (Shanghai, China); the genotypes of APC^min/+^, SR‐B1^−/+^, and SR‐B1^−/−^ mice were characterized through polymerase chain reaction (PCR). The following four primers were used for PCR analysis of DNA extracted from the tail to genotype the SR‐B1^−/+^ and SR‐B1^−/−^ mice:^15^ 5′‐AATGGACCCTGTGCTTGGAGTG‐3′, 5′‐GGAGGAGGAGGTGGTCATAGAACG‐3′, 5′‐TCCTAATCCTTCCAAGCCGTTCTC‐3′, 5′‐CAGCCATTTTGCCCATTTTGTGC‐3′. The following two primers were used for PCR analysis of DNA extracted from the tail to genotype the APC^min/+^ mice: 5′‐GTGCAGCAGCTTTAAGGAA‐3′, 5′‐AATGGAACTCGGTGGTAGA‐3′. The animals were housed in standard‐sized cages at room temperature, 24 ± 1°C, with 60% ± 5% humidity, in a 12‐h light/dark cycle and with food and water available. The Experimental Animal Management Committee of the Capital Medical University approved all studies (IACUC Protocol No: AEEI‐2019‐070). All the experiments were performed following the guidelines of the Experimental Animal Care and Use Committee of Capital Medical University (Beijing, China).

### 2.2 AOM/DSS and APC^min^ ^/+^ induced mice model

**FIGURE 1:**
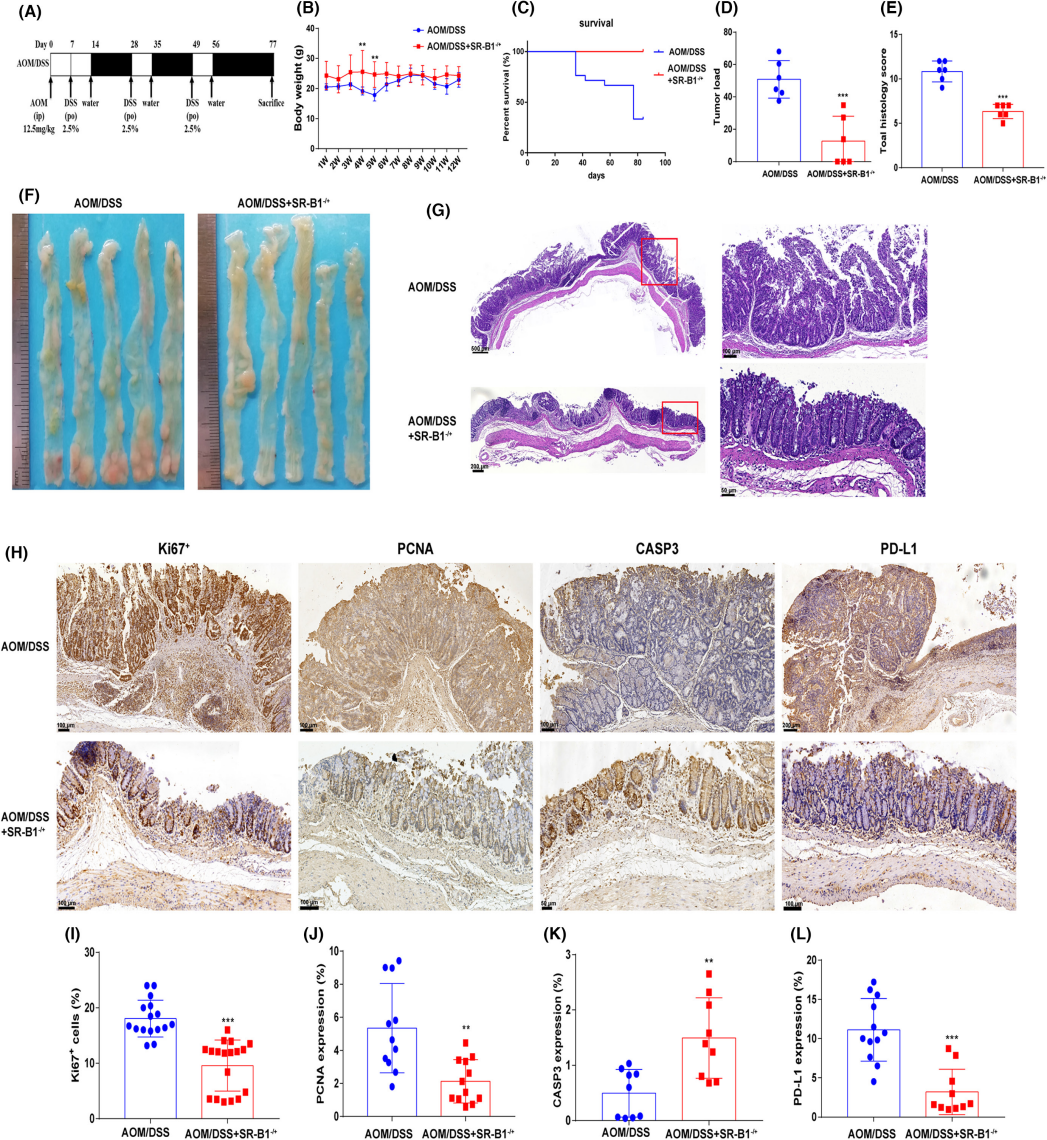
Scavenger receptor‐class B type 1 (SR‐B1) deficiency reduced the tumor load and programmed cell death‐ligand 1 (PD‐L1) level of colitis‐induced colorectal cancer (*n* = 6). The establishment scheme of the colitis‐induced colorectal cancer (CRC) model (A); the body weight changes of mice (B); the survival curve of mice (C); the tumor load of mice (D, F); the colorectal HE staining and histology scoring in mice (E, G); the Ki67^+^, PCNA, Caspase‐ 3, PD‐L1 expression by immunohistochemical staining and quantification in mice (H–L); compared with azoxymethane/dextran sodium sulfate (AOM/DSS) mice, **p < 0.01; and ***p < 0.001.

**FIGURE 2:**
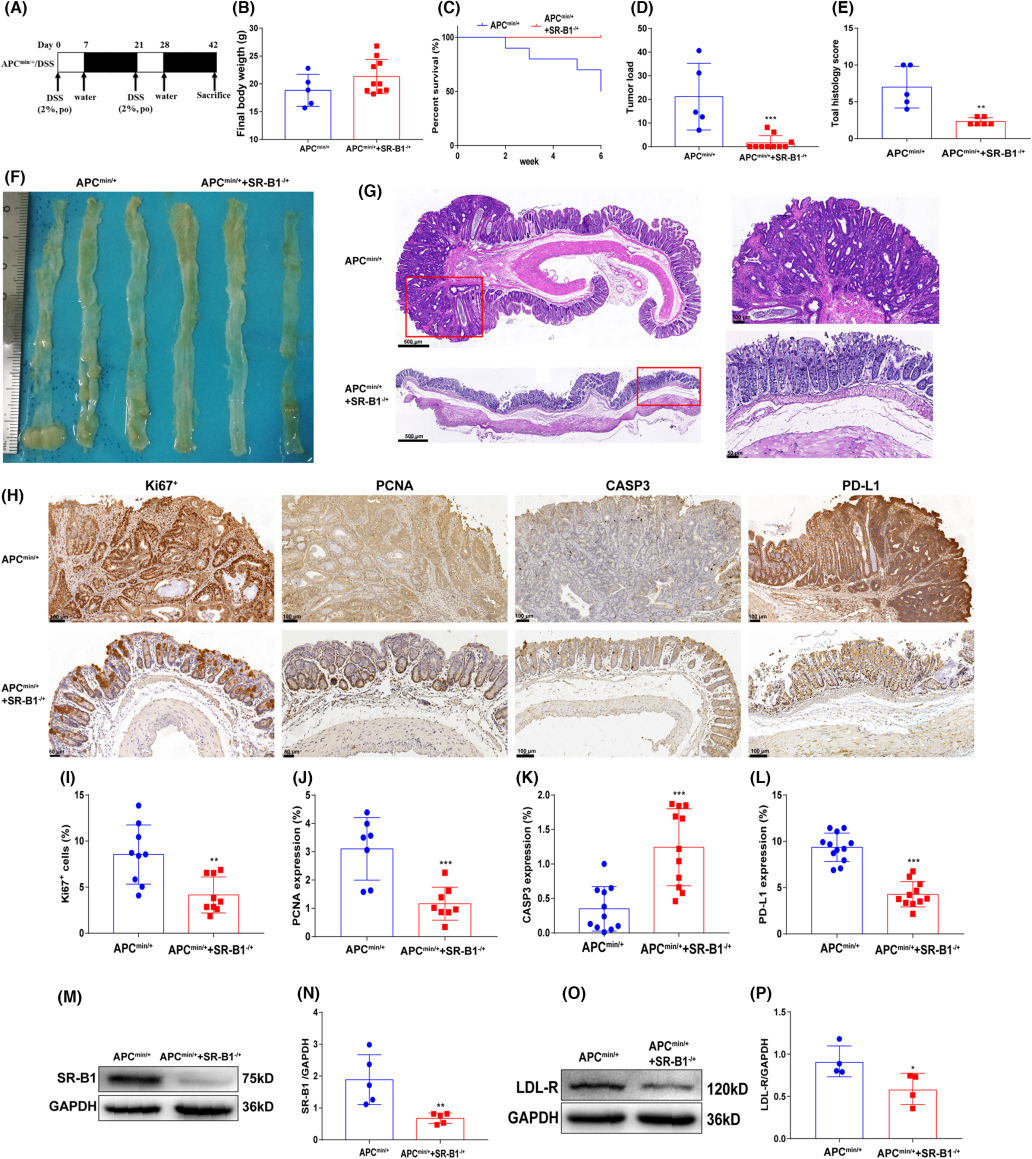
Effect of scavenger receptor‐class B type 1 (SR‐B1) gene knockdown on APC^min/+^ mice (*n* = 10). The establishment scheme of the APC^min/+^+SR‐B1^−/+^ mice CRC model (A); the body weight changes of mice (B); the survival curve of mice (C); the tumor load of mice (D, F); the colorectal hematoxylin–eosin staining and histology scoring in mice (E, G); the Ki67^+^, PCNA, Caspase‐ 3, programmed cell death‐ligand 1 (PD‐L1) expression by immunohistochemical staining and quantification in mice (H–L); the SR‐B1 and LDL‐R expression by western blot and quantification in mice (M‐P); compared with APC^min/+^, *p < 0.05, **p < 0.01; and ***p < 0.001.

The azoxymethane/dextran sodium sulfate (AOM/DSS) model was injected intraperitoneally with 12.5 mg/kg azoxymethane (AOM, 10 μL/g, A5486‐25MG, SIGMA, USA) solution on Day 1 of Week 1. After resting for 1 week, mice were fed drinking water containing 2.5% dextran sodium sulfate (DSS, MB5535, meilunbio, China) for 1 week, then switching to regular drinking water for 2 weeks and cycling three times, as shown in Figure 1A. In the AOM/DSS induced model, anti‐PD‐1 (BE0146, Bio X Cell, USA) was injected intraperitoneally on Days 50, 57, and 64, respectively, as shown in Figure 5A. The APC^min/+^ DSS model was given drinking water containing 2.0% DSS to all animals for 1 week, followed by switching to regular drinking water for 2 weeks and cycling two times, as shown in Figure 2A. We divided tumors into three groups: small tumors, <1 mm; medium tumors, 1 mm ≤ and ≤2 mm; large tumors, >2 mm. Tumor load was calculated according to the following formula: tumor load = (number of small tumors) × 1 + (number of medium tumors) × 2 + (number of large tumors) × 3.^16^

### 2.3 Immunohistochemistry and hematoxylin–eosin staining

Segments of the colorectal from the mice were fixed in freshly prepared 4% paraformaldehyde solution. Subsequently, these tissues were dehydrated in a rising series of ethanol, hyalinized in xylene, and embedded in paraffin wax. They were cut into 5 μm sections and then were routinely stained with hematoxylin–eosin (HE) and immunohistochemistry. The expression of Ki67, PCNA, CASP3, and PD‐L1 in mouse colorectal tissues was detected using the Rabbit Two‐Step Detection Kit (PV‐9001, ZSGB‐BIO, China). The mouse colorectal sections were incubated overnight at 4°C with anti‐Ki67 (1:500, K009725P, Solarbio, China), anti‐PCNA (1:500, #21338, thermo fisher, USA), anti‐CASP3 (1:500, K101291P, Solarbio, China), anti‐PD‐L1 (1:500, K009918P, Solarbio, China). Finally, the sections were scanned under Pannoramic SCAN (3DHistech, Hungary). The AOM/DSS and APC^min/+^ induced mice model histological scoring criteria follow the reference.^17^ The immunohistochemistry slice was divided into multiple regions by Image‐Pro Plus for specific antibody expression statistics, and the specific antibody expression intensity = IOD/Area.

### 2.4 Western blot analysis

The colorectal tissue whole protein was extracted using a protein lysis buffer (P0013B, Beyotime, China). 50 μg of total protein was separated by 10% SDS‐polyacrylamide gel electrophoresis and then transferred onto 0.22 μm polyvinylidene fluoride membrane. Then blocked with 5% nonfat milk powder in TBST for 2 h at room temperature and incubated with the appropriate primary antibody at 4°C overnight. The primary antibodies and their dilution concentration were as follows: anti‐SR‐B1 (1:1000, NB400‐104, Novus Biologicals, USA), anti‐LDL‐R (1:1000, K009497P, Solarbio, China), anti‐PD‐L1 (1:1000, K009918P, Solarbio, China), anti‐GAPDH (1:2500, E12‐052, EnoGene, China), anti‐ABCA1 (1:1000, ab18180, Abcam, UK), anti‐HLA‐B (1:1000, K009916P, Solarbio, China), and anti‐β‐Actin (1:2500, #21338, SAB, USA). Subsequently, the membranes were washed with 1 × TBST and incubated with secondary antibodies: Goat anti‐Mouse or anti‐Rabbit IgG (1:10000) for 1 h at room temperature. After the membranes were washed again by 1 × TBST buffer, the proteins were visualized using enhanced chemiluminescence.

### 2.5 Flow cytometry measurement of immune cell expression

Single intestinal lamina propria cells were prepared using a Miltenyi intestinal lamina propria dissociation kit (130‐095‐929, Miltenyi Biotec). Single intestinal lamina propria cells were stained with anti‐mouse CD3‐FITC, anti‐mouse NK1.1 PE‐CY7, anti‐mouse LY6C PE‐CY7, anti‐mouse CD11B‐PE, anti‐mouse CD11C‐BV421, anti‐mouse F4/80‐FITC, anti‐mouse CD45‐BV511, and anti‐mouse LY6G‐APC antibodies. The data were collected with a Fortessa Flow Cytometer (BD Biosciences, San Jose, CA) and analyzed with FlowJo version 10 software (Tree Star Inc., Ashland, OR).

### 2.6 Eukaryotic mRNA sequencing

Eukaryotic mRNA sequencing is based on the Illumina Novaseq 6000 sequencing platform and uses the Illumina TruseqTM RNA sample prep kit method for library construction. Total RNA was extracted from APC^min/+^ + SR‐B1^−/+^ and APC^min/+^ mice colorectal tissue, enriched by Oligo dT, then fragmented mRNA, inverted to synthesize cDNA, and ligated adaptor, and finally sequenced on the Illumina platform. The raw data of transcriptome sequencing were filtered to screen for differentially expressed genes. The KEGG annotation and enrichment analysis and protein–protein interaction analysis were applied to determine the roles of these differentially expressed mRNAs in biological pathways.

### 2.7 16S rRNA high‐throughput sequencing

Bacterial DNA was extracted and purified from each fecal sample with an E.Z.N.A.® Soil DNA Kit (Omega Bio‐Tek, Norcross, GA, USA). The V3‐V4 region of the bacterial 16S rRNA gene was PCR amplified with primers 338F and 806R. The sequencing libraries were prepared using a TruSeq Nano DNA LT Library Prep Kit of Illumina. Library quality testing was performed with an Agilent High Sensitivity DNA kit and 2 × 300 bp double‐ended sequencing of 2 × 300 bp using a MiSeq 200–450 bp sequencer. The effective sequences were normalized and classified into operational taxonomies (OTUs) based on 97% sequence similarity with QIIME 2 software. The most abundant sequence in each OTU is considered the representative sequence of the OTU. The representative OTU sequences were compared with template sequences in the Greengenes database (Release 13.8) to obtain the bacterial count information of each OTU based on six classification levels: phyla, class, order, family, genus, and species. The fecal gut microbiome was analyzed by taxonomic composition analysis, alpha and beta diversity analysis, and other advanced methods.

### 2.8 Statistical analysis

GraphPad Prism 8.0.1, QIIME 2 and R software packages (v3.2.0) were used for statistical analysis. The data are expressed as the means ± standard deviation (SD). Student's *t*‐test was performed to compare two groups, one‐way ANOVA was performed to compare multiple groups, and the abundance of gut microbiota was compared by Kruskal–Wallis rank sum test.

## 3 RESULTS

### 3.1 SR‐B1 deficiency reduced the tumor load and PD‐L1 level of colitis‐induced CRC

As shown in Figure 1, the body weight of AOM/DSS + SR‐B1^−/+^ mice was significantly higher than that of AOM/DSS mice at weeks 4 and 5 (*p* < 0.05). At the end of the animal experiment, the AOM/DSS mice had severe blood in the stool and prolapse. The mortality rate of AOM/DSS mice was 66.7%, while the mortality rate of AOM/DSS + SR‐B1^−/+^ mice was 0. Compared with AOM/DSS mice, the tumor load of AOM/DSS + SR‐B1^−/+^ mice was significantly reduced (*p* < 0.01). The HE staining showed that AOM/DSS mice had more inflammatory cell infiltration, crypt aberrations, and heterosexual hyperplasia in the colorectum. The immunohistochemical staining showed that compared with the AOM/DSS mice, the level of Ki67^+^, PCNA in AOM/DSS + SR‐B1^−/+^ mice was significantly reduced, while the level of CASP3 was significantly increased (*p* < 0.01), which indicated that SR‐B1 knockdown inhibits the growth of tumor cells. Furthermore, the expression of PD‐L1 in AOM/DSS + SR‐B1^−/+^ mice was lower than that of AOM/DSS mice (*p* < 0.001).

### 3.2 SR‐B1 deficiency reduced the tumor load and PD‐L1 level of APC^min^ ^/+^‐induced CRC

As shown in Figure 2, there was no significant difference in the final weight of APC^min/+^+SR‐B1^−/+^ mice and APC^min/+^ mice. The APC^min/+^ mice have more blood in the colorectal stool, the mortality rate of APC^min/+^ mice was 50%, and the mortality rate of APC^min/+^+SR‐B1^−/+^ mice was 0. Compared with the APC^min/+^ mice, the tumor load of APC^min/+^+SR‐B1^−/+^ mice was significantly reduced (*p* < 0.001). The HE staining showed that the colorectal inflammatory infiltration of APC^min/+^ mice was more serious, and the histology score of APC^min/+^ mice was significantly higher than that of APC^min/+^+SR‐B1^−/+^ mice (*p* < 0.01). The immunohistochemical staining showed that compared with APC^min/+^ mice, the level of Ki67^+^, PCNA in APC^min/+^+SR‐B1^−/+^ mice was significantly reduced, while the level of CASP3 was significantly increased (*p* < 0.01), which indicated that SR‐B1 knockdown inhibits the growth of tumor cells. Moreover, compared with the APC^min/+^ mice, the expression of PD‐L1 in APC^min/+^+SR‐B1^−/+^ mice reduced significantly (*p* < 0.001). The western blot shown that compared with the APC^min/+^ mice, the expression of SR‐B1, LDL‐R in APC^min/+^+SR‐B1^−/+^ mice colorectal tissue reduced significantly (*p* < 0.05).

### 3.3 SR‐B1 deficiency regulated the expression of genes in APC^min^ ^/+^‐induced CRC

**FIGURE 3:**
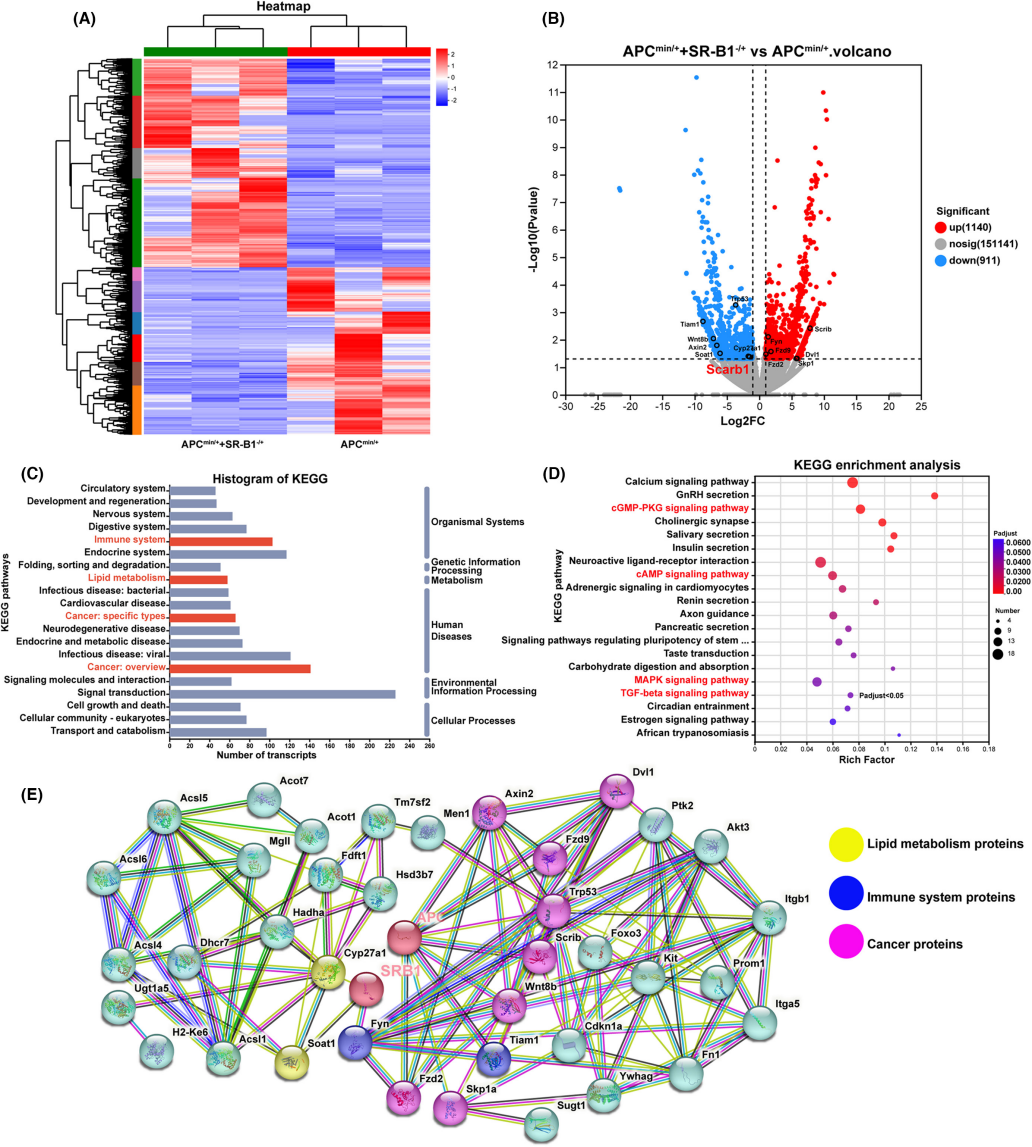
Scavenger receptor‐class B type 1 (SR‐B1) deficiency regulated the expression of genes in APC^min/+^‐induced colorectal cancer (*n* = 3). The gene transcripts cluster analysis (A); the gene transcripts expression difference statistics (B); the KEGG annotation analysis (C); the KEGG enrichment analysis (D); the protein–protein interaction analysis (E); *p*_adjust_ <0.05.

As shown in Figure 3, the cluster analysis showed significant differences in gene transcripts of APC^min/+^+SR‐B1^−/+^ mice and APC^min/+^ mice. The volcano showed that APC^min/+^+SR‐B1^−/+^ mice significantly upregulated 1140 gene transcripts and significantly downregulated 911 gene transcripts. The KEGG annotation analysis found that APC^min/+^+SR‐B1^−/+^ mice regulate the expression of genes associated with the immune system, lipid metabolism, and cancer. The results of KEGG enrichment analysis showed significant differences between APC^min/+^+SR‐B1^−/+^ mice and APC^min/+^ mice in pathways such as TGF‐beta signaling pathway, MAPK signaling pathway, cAMP signaling pathway, and cGMP‐PKG signaling pathway (*p* < 0.05). These pathways regulate multiple physiological processes such as cell growth, differentiation, apoptosis, and death, and the TGF‐beta signaling pathway plays a key role in immune function and inflammatory response. The protein–protein interaction analysis found that SR‐B1 protein may downregulate the expression of lipid metabolism‐related proteins such as Cyp27a1 and Soat1, and may downregulate the expression of Tiam1 and upregulate the expression of Fyn of immune system‐related proteins. APC protein may have downregulated the expression of Wnt8b, Trp53, Axin2, and upregulated the expression of Fzd2, Skp1a, Scrib, Fzd9, Dvl1 of tumor‐associated proteins. Therefore, SR‐B1 protein may interact with APC protein through the lipid metabolism, immune systems, and tumor‐associated proteins.

### 3.4 SR‐B1 deficiency controlled the intestinal immunity cell level levels in colitis‐induced CRC

**FIGURE 4:**
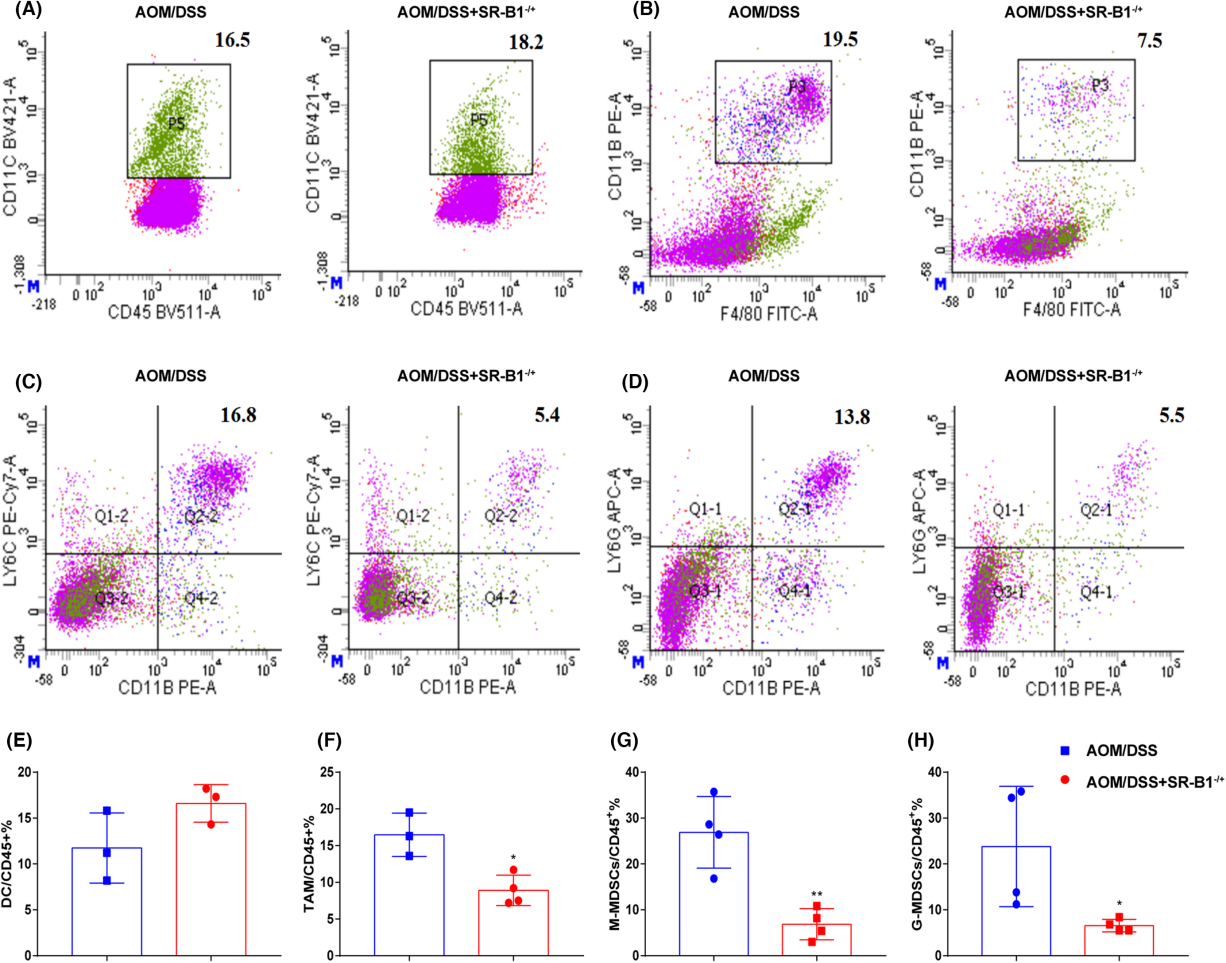
Scavenger receptor‐class B type 1 (SR‐B1) deficiency affected intestinal immunity in colitis‐induced colorectal cancer (*n* = 3–4). Flow cytometry detected the levels of dendritic cells (DCs) (A, E), tumor‐associated macrophage (TAM) (B, F), mononuclear myeloid‐derived suppressor cells (M‐MDSCs) (C, G), and granulocytic myeloid‐derived suppressor cells (G‐MDSCs) (D, H) in colorectal tissue of SR‐B1^−/+^ mice; compared with azoxymethane/dextran sodium sulfate (AOM/DSS) mice, *p < 0.05, **p < 0.01.

As shown in Figure 4, compared with AOM/DSS mice, the level of DCs in AOM/DSS + SR‐B1^−/+^ mice had elevated, but there was no significant difference. MDSCs are bone marrow‐derived inhibitory cells, precursors of dendritic cells, macrophages, and granulocytes, and we found that the levels of TAM, M‐MDSCs, and G‐MDSCs in AOM/DSS + SR‐B1^−/+^ mice were significantly reduced compared with AOM/DSS mice (*p* < 0.05), indicating that AOM/DSS + SR‐B1^−/+^ mice reduced the ability to suppress immune cell responses.

### 3.5 SR‐B1 deficiency triggered the anti‐colon effect of anti‐PD‐1 in colitis‐induced CRC

**FIGURE 5:**
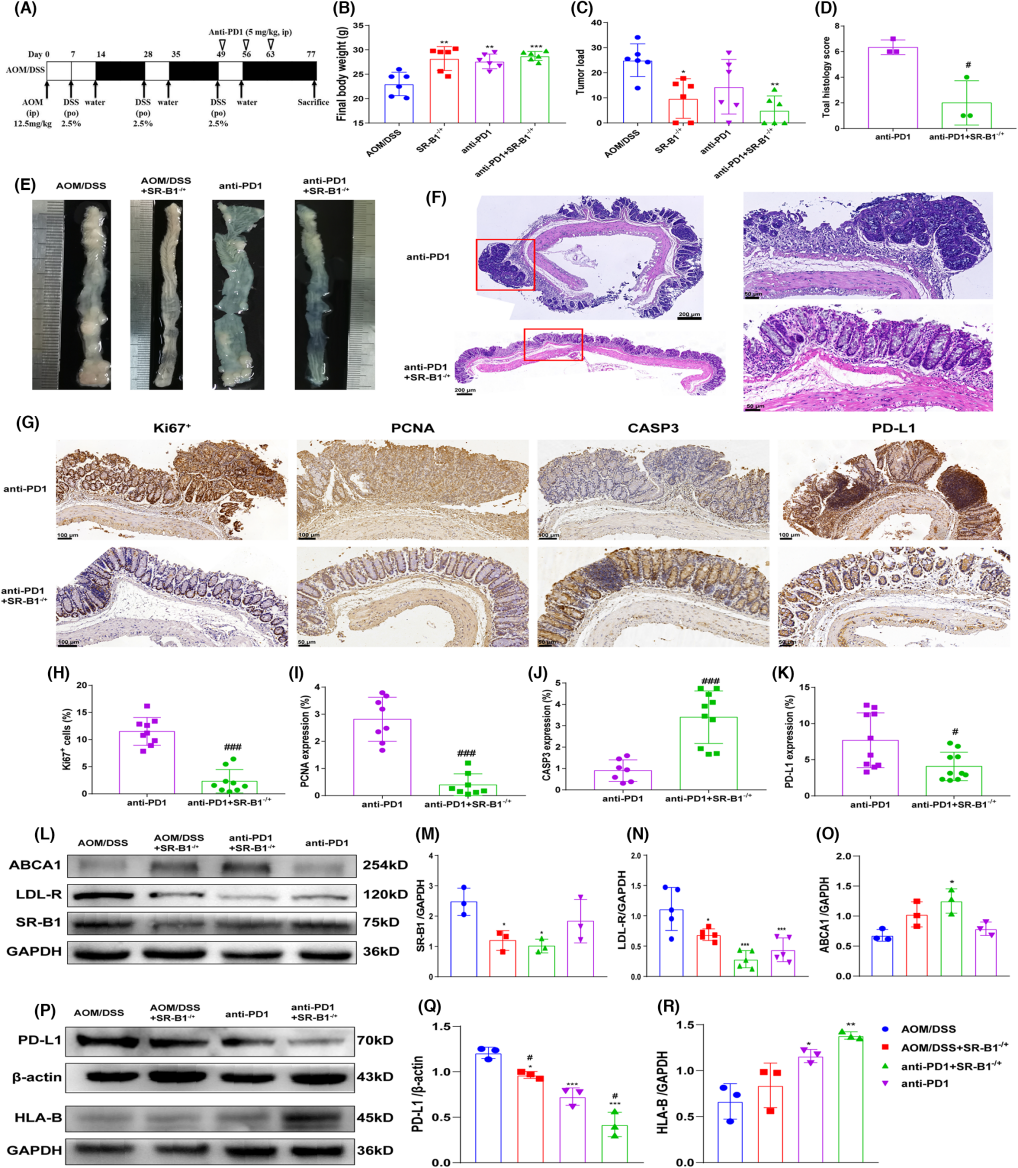
Scavenger receptor‐class B type 1 (SR‐B1) deficiency triggered the anti‐colon effect of anti‐PD‐1 in colitis‐induced colorectal cancer (CRC) (*n* = 6). The CRC model and anti‐PD1 treatment program (A); the final body weight of mice (B); he tumor load of mice (C, E); the HE staining and histology scoring in mice (D, F); the colorectal Ki67^+^, PCNA, Caspase‐ 3, programmed cell death‐ligand 1 (PD‐L1) expression by immunohistochemical staining and quantification in mice (G–K); the SR‐B1, LDL‐R, ABCA1 (L‐O), PD‐L1, HLA‐B (P–R) expression by western blot and quantification in mice; compared with azoxymethane/dextran sodium sulfate (AOM/DSS) mice, *p < 0.05, **p < 0.01; and ***p < 0.001; compared with anti‐PD1 mice, ^#^ *p* < 0.05, and ^###^ *p* < 0.001.

As shown in Figure 5, compared with AOM/DSS mice, the AOM/DSS + SR‐B1^−/+^, anti‐PD1, anti‐PD1 + SR‐B1^−/+^ mice had a significantly higher final body weight (*p* < 0.01), and the tumor load in AOM/DSS + SR‐B1^−/+^ and anti‐PD1 + SR‐B1^−/+^ mice was significantly reduced (*p* < 0.05). The HE staining showed that anti‐PD1 mice had severe colorectal inflammatory infiltration, crypt aberration, intrinsic layers hyperplasia, and the histology score of anti‐PD1 mice was significantly higher than that of anti‐PD1 + SR‐B1^−/+^ mice (*p* < 0.05). The immunohistochemical staining showed that compared with anti‐PD1 mice, the level of Ki67^+^, PCNA in anti‐PD1 + SR‐B1^−/+^ mice were significantly reduced, while the level of CASP3 was significantly increased (*p* < 0.001), which indicated that SRB1 knockdown combined with anti‐PD1 therapy significantly inhibits the growth of tumor cells. Compared with the anti‐PD1 mice, the expression of PD‐L1 in anti‐PD1 + SR‐B1^−/+^ mice was reduced significantly (*p* < 0.05). The western blot shown that compared with the AOM/DSS mice, the expression of SR‐B1, LDL‐R in AOM/DSS + SR‐B1^−/+^, anti‐PD1 + SR‐B1^−/+^ mice colorectal tissue reduced significantly (*p* < 0.05), while the expression of ABCA1 in anti‐PD1 + SR‐B1^−/+^ mice colorectal tissue increased significantly (*p* < 0.05). Compared with the AOM/DSS mice, the expression of PD‐L1 in AOM/DSS + SR‐B1^−/+^, anti‐PD1, and anti‐PD1 + SR‐B1^−/+^ mice colorectal tissue reduced significantly (*p* < 0.05), while the expression of HLA‐B in anti‐PD1 and anti‐PD1 + SR‐B1^−/+^ mice colorectal tissue increased significantly (*p* < 0.05).

### 3.6 SR‐B1 deficiency regulated the intestinal microbiota of colitis‐induced CRC

**FIGURE 6:**
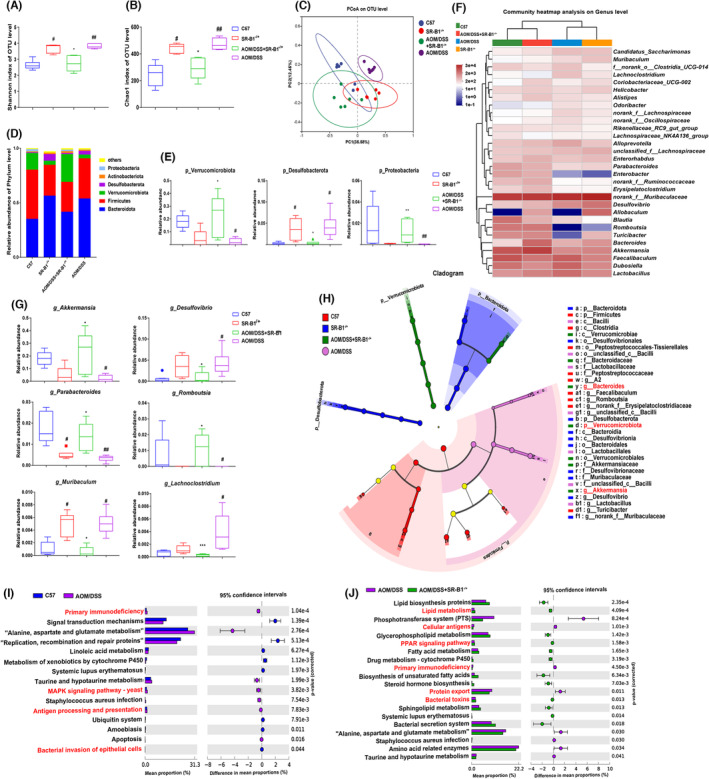
Scavenger receptor‐class B type 1 (SR‐B1) deficiency regulated the intestinal microbiota of colitis‐induced colorectal cancer mice (*n* = 6). The Shannon (A) and CHAO1 (B) index of alpha diversity analysis; principal coordinate analysis (PCoA) of the beta diversity analysis results (C); the relative abundance of the gut microbiota (D) and significantly changed gut microbiota (E) at the phylum; the heatmap analysis of gut microbiota (F) and significantly changed gut microbiota (G) on genus level; the Lefse analysis (H); predicted functions of the altered metagenome in the fecal microbiota of the AOM/DSS + SR‐B1^−/−^ mice, as indicated by Kyoto Encyclopedia of Genes and Genomes (KEGG) pathway analysis; only pathways enriched with a *p* value <0.05 are shown (I, J); *p < 0.05, **p < 0.01; and ***p < 0.001 compared with the AOM/DSS mice; ^#^ *p* < 0.05, ^##^ *p* < 0.01; compared with the C57 mice.

As shown in Figure 6, the results of α diversity analysis showed that the Shannon and CHAO1 index of AOM/DSS, SR‐B1^−/+^ mice were significantly increased compared with C57 mice (*p* < 0.05), while that of AOM/DSS + SR‐B1^−/+^ mice were significantly reduced compared with AOM/DSS mice (*p* < 0.05). It indicated that the intestinal microbiota richness and diversity of AOM/DSS mice were significantly increased. The β diversity analysis of PCoA showed that the intestinal microbiota structure of AOM/DSS mice was significantly isolated from C57, SR‐B1^−/+^ and AOM/DSS + SR‐B1^−/+^ mice, while the intestinal microbiota structure of C57, SR‐B1^−/+^, and AOM/DSS + SR‐B1^−/+^ mice was similar.

Taxonomic analysis at the phylum level showed that Bacteroidota and Firmicutes were the most abundant in the gut microbiota. Compared with C57 mice, the abundance of Verrucomicrobiota and Proteobacteria was significantly reduced (*p* < 0.05), while the abundance of Desulfobacterota was significantly increased in AOM/DSS mice (*p* < 0.05). Compared with AOM/DSS mice, the abundance of Verrucomicrobiota and Proteobacteria was significantly increased (*p* < 0.05), while the abundance of Desulfobacterota was significantly reduced in AOM/DSS + SR‐B1^−/+^ mice (*p* < 0.05). At the genus level, compared with C57 mice, the abundances of *Akkermansia*, *Parabacteroides* and *Romboutsia* were significantly reduced (*p* < 0.05), while the abundance of *Desulfovibrio*, *Muribaculum*, and *Lachnoclostridium* was significantly increased in AOM/DSS mice (*p* < 0.05). Compared with AOM/DSS mice, the abundances of *Akkermansia*, *Parabacteroides* and *Romboutsia* were significantly increased (*p* < 0.05), while the abundance of *Desulfovibrio*, *Muribaculum*, and *Lachnoclostridium* was significantly reduced in AOM/DSS + SR‐B1^−/+^ mice (*p* < 0.05).

The results of the KEGG pathway analysis showed significant differences between AOM/DSS and C57 mice in pathways such as primary immunodeficiency, MAPK signaling pathway—yeast, antigen processing and presentation, and bacterial invasion of epithelial cells (*p* < 0.05). The pathways of lipid metabolism, cellular antigens, PPAR signaling pathway, primary immunodeficiency, protein export, and bacterial toxins were significant differences between AOM/DSS and AOM/DSS + SR‐B1^−/+^ mice (*p* < 0.05).

## 4 DISCUSSION

SR‐B1 deficiency reduced the tumor load and PD‐L1 level of colitis‐induced or APC^min/+^‐induced CRC, triggered the anti‐colon effect of anti‐PD‐1 in colitis‐induced CRC, which controlled the TAM, M‐MDSCs, G‐MDSCs, PD‐L1, and increased the HLA‐B levelin colitis‐induced CRC, regulated the expression of genes in APC^min/+^‐induced CRC, and regulated the intestinal microbiota of colitis‐induced CRC. SR‐B1 exerted anti‐CRC effects by regulating the immune system, cholesterol metabolism, and gut microbiota. In addition, SR‐B1 deficiency triggered the anti‐CRC effect of anti‐PD‐1 in colitis‐induced CRC. Moreover, SR‐B1 deficiency reduced the LDL‐R level and increased the ABCA1 level of colitis‐induced CRC.

Scavenger receptors are highly expressed in tumors and are associated with prognosis. Targeting scavenger receptor MARCO alters macrophage polarization and activates NK cells in the tumor.^18^ The high expression of SR‐A is significantly related to the invasion and metastasis of colon cancer.^19^ In this study, SR‐B1 deficiency reduced the tumor load of colitis‐induced or APC^min/+^‐induced CRC, triggered the anti‐colon effect. This study provides a new treatment strategy for colore cancer. Furthermore, scavenger receptors are closely related to immune regulation and antigen presentation. Scavenger receptors CD163, SREC‐I, SR‐AI/II, MARCO, and CD36 regulate innate immunity. CD163 on macrophages acted as an innate immune sensor and an inducer of local inflammation during bacterial infection.^20^ SREC‐I modulated the function of toll‐like receptors with a significant role in CD8^+^‐ and CD4^+^‐mediated T‐cell immunity.^21^, ^22^ SR‐A was required for NF‐κB response activation in macrophages,^23^ mediated the cross‐presentation of antigens, and regulated anti‐tumor immune consequences.^24^, ^25^ MARCO mediated macrophage polarization of immune responses.^21^ CD36 was also related to MHC class II antigen presentation.^26^ In this study, SR‐B1 deficiency controlled the TAM, M‐MDSCs and G‐MDSCs and PD‐L1 level and increased the level of HLA‐B in the intestine of the colitis‐induced CRC mice. Our results further support the critical role of scavenger receptors in regulating the anti‐tumor immune response. SR‐B1 deficiency regulates the expression of genes associated with the immune system, lipid metabolism and cancer. In addition, PD‐1 blockade has shown clinical benefits. Studies showed a synergistic effect when CD73 was combined with PD‐1 blocker immunotherapy.^27^ Combination therapy with anti‐CTL‐A4/anti‐PD‐1 monoclonal antibody therapy for CRC has shown lasting clinical benefit and acceptable safety.^28^ Our study showed that SR‐B1 knockout could trigger the anti‐colon effect of anti‐PD 1 in colitis‐induced CRC.

More notably, as the tumor microenvironment of colon cancer, intestinal microbiota was closely related to the occurrence and development of colon cancer.^29^, ^30^ *Akkermansiaceae*, *Parabacteroides*, and *Romboutsia* were beneficial bacteria for colorectal cancer patients, while *Desulfovibrio*, *Muribaculum*, and *Lachnoclostridium* were just the opposite. *Akkermansiaceae Muciniphila* was one of the immunotherapy‐inducing microbiota.^31^ It was favorable for immune response to chemotherapeutic drugs and anti‐programmed death protein 1 (PD‐1) therapy.^32^, ^33^ *Parabacteroides* elevated intestinal barrier integrity in azoxymethane‐induced colorectal tumorigenesis.^34^ *Romboutsia* was one of the most beneficial probiotics in CRC. *Romboutsia* was found to be significantly poor in CRC, which was a valuable biomarker for preventing the development of CRC.^35^ *Lachnoclostridium* was significantly abundant in colorectal adenoma, and *Lachnoclostridium* sp. was significantly increased in patients with CRC.^36^, ^37^ So *Lachnoclostridium* was used as an early diagnostic biomarker of CRC. The *Muribaculum* genus was significantly enriched in AOM/DSS induced CRC and T cell‐induced colitis mice.^38^, ^39^ Furthermore, *Desulfovibrio*, one of the sulfidogenic bacteria, participated in the progress of CRC.^40^, ^41^, ^42^ In this study, SR‐BI deficiency reduced the tumor load, and the abundances of *Akkermansiaceae*, *Parabacteroides*, and *Romboutsia* were significantly increased in AOM/DSS+SR‐B1^−/+^ mice; the abundances of *Desulfovibrio*, *Muribaculum*, and *Lachnoclostridium* were apparently reduced. Our experimental results were consistent with the antagonistic effect of *Akkermansiaceae*, *Parabacteroides*, and *Romboutsia* and the promoted effect of *Desulfovibrio*, *Muribaculum*, and *Lachnoclostridium* on intestinal tumors. In addition, the gut microbiome can serve as a potential weapon to optimize immune checkpoint inhibitor immunotherapy. Indole‐3‐carboxaldehyde, a microbial tryptophan catabolite, protected mice from intestinal damage via a dual action on both the host and the microbes.^43^ The results of the KEGG pathway analysis showed that the MAPK signaling pathway—yeast, antigen processing, and presentation were significant differences between AOM/DSS and C57 mice, and the PPAR signaling pathway and cellular antigens were significant differences between AOM/DSS and AOM/DSS + SR‐B1^−/+^ mice. The mechanism of action of SR‐B1 deficiency on the development of CRC still needs further in‐depth research.

In conclusion, SR‐B1 knockdown reduced the tumor load of colitis‐induced or APC^min/+^‐induced CRC. SR‐B1 knockdown improved the immune microenvironment by controlling the TAM, M‐MDSCs and G‐MDSCs, PD‐L1 and HLA‐B levels to improve the immune microenvironment, and reduce the level of LDL‐R, and increase the level of ABCA1 to regulate the cholesterol metabolism, and regulated the expression of genes and intestinal microbiota. SR‐B1 knockdown can also trigger the anti‐colon effect of anti‐PD 1 in colitis‐induced CRC. This study provides a new treatment strategy for SR‐B1 in colorectal cancer.

## AUTHOR CONTRIBUTIONS

**Qijun Chen:** Data curation (equal); formal analysis (equal); investigation (equal); methodology (equal); software (equal); validation (equal); writing – original draft (equal). **Lixue Wang:** Data curation (equal); formal analysis (equal); investigation (equal); methodology (equal); software (equal); supervision (equal); validation (equal); visualization (equal). **Hui Song:** Investigation (equal); methodology (equal); resources (equal); software (equal); validation (equal). **Wen Xing:** Formal analysis (equal); resources (equal); software (equal); supervision (equal); validation (equal); visualization (equal). **Junfeng Shi:** Data curation (equal); formal analysis (equal); investigation (equal); methodology (equal); resources (equal); software (equal); supervision (equal); validation (equal); visualization (equal). **Yudi Li:** Formal analysis (equal); resources (equal); software (equal); supervision (equal); validation (equal); visualization (equal). **Ziqian Wang:** Methodology (equal); software (equal); supervision (equal); visualization (equal). **Jinlong Chen:** Investigation (equal); methodology (equal); supervision (equal); validation (equal); visualization (equal). **Nan Xie:** Formal analysis (equal); investigation (equal); validation (equal); visualization (equal). **Wenhua Zhao:** Conceptualization (equal); funding acquisition (equal); project administration (equal); writing – original draft (equal); writing – review and editing (equal).

## FUNDING INFORMATION

This work was supported by the National Science Foundation of China (81973442, ZWH), the Scientific Research Common Program of Beijing Municipal Commission of Education (KM201910025022, ZWH), and the Importation and Development of High‐Caliber Talents Project of Beijing Municipal Institutions (CIT&TCD201304176, ZWH).

## CONFLICT OF INTEREST STATEMENT

The authors have no conflict of interest.

## ETHICS STATEMENT

Our animal studies were approved by the Experimental Animal Management Committee at Capital Medical University (IACUC Protocol No: AEEI‐2019‐070). All experimental procedures were carried out following the guidelines of the Experimental Animal Care and Use Committee of Capital Medical University.

## Data Availability

The datasets used and/or analyzed during the current study are available from the corresponding author upon reasonable request.
